# Functional role and implications of population heterogeneity on vestibular ocular reflex response fidelity

**DOI:** 10.1186/1471-2202-14-S1-P147

**Published:** 2013-07-08

**Authors:** James McGuinness, Bruce P Graham

**Affiliations:** 1 Computing Science and Mathematics, School of Natural Sciences, University of Stirling, Stirling, FK9 4LA, UK

## 

Evidence for a functional role of population variance and heterogeneity in sensori-motor systems is currently emerging [1]. The vestibular-ocular reflex (VOR) is a sensori-motor reflex characterised by high fidelity, low latency compensatory eye movements in response to head movements. A major component of the VOR is the linear, rotational reflex, which produces eye movements in order to compensate for angular head rotations [2]. This linear, rotational component of the VOR is produced through an elementary 3 neuron arc. However, single cell responses of the vestibular nucleus neurons involved show non-linearity and distortion for high frequency inputs [3] suggesting, instead, that the required signal transmission is achieved by response of a population of neurons [4].

Using biophysically complete, compartmental models of medial vestibular nucleus (MVN) neurons, utilising the Hodgkin-Huxley formulation applied to a range of ionic currents, we investigate the effects of variance and heterogeneity of ion channel density in producing and maintaining asynchronicity of the population, and the effect of this on the response fidelity of the population to a broad range of input frequencies. Inputs used take the form of both sinusoidal current injection, as well as driving of the cell model through synaptic input.

It will be shown that heterogeneous populations, where each member shows a different rate of spontaneous activity, produces a response with greater fidelity than homogeneous populations, in which firing regularity is the same across the population. This heterogeneity in the populations is produced through the variance of calcium activated potassium channels, present in both the somatic and dendritic compartments of the model, involved in the second phase of a biphasic action potential. The ranges and distributions of variance across populations will be discussed in terms of optimisation and tuning of the circuits involved in the response.

In addition, various commonly used methods for the analysis and estimation of the population response are discussed in regards to their suitability, accuracy and inherent limitations. Finally, techniques for the analysis involving the use of optimal digital filters will be discussed.
